# Supplementary material for: Time-dependent cytokine and chemokine changes in mouse cerebral cortex following a mild traumatic brain injury
Source: eLife. 2020 Aug 17;9:e55827. doi: 10.7554/eLife.55827 (PMC7473773; doi:10.7554/eLife.55827)
Supplement: Supplementary file 1. — Provided are lists of up-regulated and down-regulated proteins, also the mean, SEM and number of values for each time point. Data are expressed a percentage increase from sham and complement the source data files. [file elife-55827-supp1.docx]

**Supplementary File 1 Proteins significantly regulated by a single mTBI event over time**

Proteins significantly regulated in cortical tissue on 8 hours post mTBI

Data are presented as percent 'increase' from sham.

*Up regulated Down regulated*

**Protein mean sem n Protein mean sem n**

MIP-3b 173 63 7 FASL -30 8 12

SLAM 168 46 8 MIP-1a -58 10 9

VEGF-B 142 50 8

CRP 104 23 12

IL-33 70 26 10

Fcg RIIB 53 16 12

Eotaxin-2 50 14 11

IFNg R1 49 12 12

MIP-1 gamma 47 20 12

MMP-2 46 16 12

Chemerin 45 16 12

MBL-2 44 17 12

TROY 42 8 12

gp130 39 11 12

GAS 1 36 9 12

Testican 3 35 8 12

BTC 34 10 12

DAN 25 3 12

PDGF-AA 23 4 12

Lungkine 22 9 9

bFGF 18 4 12

Progranulin 16 3 12

Proteins significantly regulated in cortical tissue on 24 hours post mTBI

Data are presented as percent 'increase' from sham.

*Up regulated Down regulated*

**Protein mean sem n Protein mean sem n**

CD6 89 28 9 Periostin -16 4 11

E-Cadherin 69 15 8 Adiponectin -18 2 12

Galectin-3 56 11 12 Fractalkine -19 4 12

CD27 55 19 12 Neprilysin -19 3 12

VEGF-B 39 13 7 JAM-A -19 7 12

GAS 1 26 6 12 MIG -20 3 12

Decorin 21 6 12 TCK-1 -23 5 12

bFGF 20 7 12 IGFBP-6 -23 4 12

Lungkine 20 7 12 Dtk -24 7 12

MCSF 17 6 12 IL-4 -25 6 12

Galectin-1 16 2 12 IL-10 -29 6 12

IL-15 -29 5 12

BLC -29 3 12

TCA-3 -31 5 12

IL-1b -32 7 10

IFN-ɣ -35 4 3

CXCL16 -36 3 11

GITR -36 7 11

TWEAK R -37 13 12

CD36 -38 11 11

IL-6 -38 5 9

MFG-E8 -39 3 12

Eotaxin -41 9 12

IL-17 -42 7 8

CD30 L -44 9 12

IL-5 -44 8 7

Granzyme B -48 12 6

E-Selectin -49 2 5

IL-20 -49 10 9

Artemin -49 6 7

FAS L -50 9 12

IL-1 R4 -52 9 12

IL-13 -54 12 8

IL-7 -55 11 11

Chemerin -55 4 10

Galectin-7 -55 4 11

TECK -56 10 7

ADAMT S1 -58 13 7

MMP-10 -60 8 11

G-CSF -62 10 7

THPO -62 6 3

MIP-1a -66 5 11

HAI-1 -68 10 4

MCP-5 -73 5 5

IL-12p40 -75 5 8

IL-1 ra -75 4 6

Proteins significantly regulated in cortical tissue on 48 hours post mTBI

Data are presented as percent 'increase' from sham.

*Up regulated Down regulated*

**Protein mean sem n Protein mean sem n**

OPN 143 56 8 Neprilysin -19 7 8

CRP 135 56 8 IL-2 Ra -22 2 8

E-Cadherin 103 18 8 IGFBP-2 -26 5 7

IL-17 E 102 32 8 IL-15 -29 12 8

CD48 96 29 8 ANG-3 -31 8 8

Renin 1 93 47 8 JAM-A -33 6 8

OPG 90 19 8 GITR -40 7 8

CD40 L 79 27 7 Fractalkine -40 4 8

Lipocalin-2 70 22 8 RANTES -41 3 3

BAFF R 66 19 7 Pro-MMp-9 -42 7 8

Tryptase E 66 11 7 TCA-3 -42 8 8

MBL-2 60 17 8 CXCL16 -43 7 8

OX40 Ligand 54 22 8 Artemin -45 10 6

Clusterin 39 6 8 Endoglin -46 5 8

GAS 1 38 6 8 IL-1 ra -48 14 7

TNF-R2 37 7 8 MFG-E8 -48 6 7

Flt-3L 36 8 7 MMP-10 -49 16 7

ACE 36 15 8 GITR L -55 9 7

bFGF 33 7 8 RAGE -57 7 5

Progranulin 32 9 8 TECK -61 8 5

DECORIN 30 9 8 FAS L -67 5 8

NOV 22 8 8 Pentraxin 3 -68 4 4

Galectin-1 15 5 8 ADAMT S1 -72 1 3

TWEAK R -73 5 7

IL-1 R4 -74 5 8

sFRP-3 -75 3 8

MadCAM-1 -86 3 3

Proteins significantly regulated in cortical tissue on 72 hours post mTBI

Data are presented as percent 'increase' from sham.

*Up regulated Down regulated*

**Protein mean sem n Protein mean sem n**

CRP 84 29 12 IL-2 RA -21 7 12

IL-17E 83 14 12 MIG -22 8 12

VEGF-B 82 26 9 Fractalkine -22 7 12

GAS 1 42 4 11 ANG-3 -23 6 11

TNF-R2 37 12 12 IFN-g -25 10 9

BTC 32 11 12 Galectin-7 -26 7 12

ACE 27 10 12 IL-1b -27 5 11

gp130 25 5 12 IL-1 R4 -28 10 12

Progranulin 23 7 12 IL-6 -31 10 10

CXCL16 -32 5 12

TCA-3 -33 5 12

RAGE -41 9 10

GITR -42 7 11

FAS L -46 5 11

Eotaxin-2 -46 11 9

RANTES -48 14 9

P-Cadherin -63 8 7

TGFb1 -65 11 7

Granzyme B -85 3 4

TRANCE -86 5 4

G-CSF -88 4 4

Proteins significantly regulated in cortical tissue on 96 hours post mTBI

Data are presented as percent 'increase' from sham.

*Up regulated Down regulated*

**Protein mean sem n Protein mean sem n**

IL-20 160 18 7 MCSF -19 7 8

VEGF-B 159 37 8 MIG -26 8 8

CD27 150 15 7 JAM-A -28 2 8

IL-17 E 131 40 8 IGFBP-2 -29 4 8

CRP 99 25 8 HGF -32 7 8

I-TAC 70 13 8 CXCL16 -34 5 8

E-Cadherin 60 20 8 MIP-1G -39 5 8

Galectin-3 50 16 8 Periostin -43 5 7

MBL-2 46 10 8 Fractalkine -45 4 8

Gas 1 46 8 8 CD30 L -46 6 8

Tryptase E 45 9 8 FAS L -46 10 8

BAFF R 38 7 8 IL-1 R4 -47 3 8

Trail 36 14 8 IL-1 ra -47 12 8

Dtk 33 10 8 MIP-1a -48 5 7

Marapsin 22 4 7 IL-9 -48 6 8

Progranulin 20 4 7 Endoglin -49 3 8

Pentraxin 3 -53 9 8

TACI -53 6 5

Eotaxin-2 -56 9 6

RANTES -58 15 5

sFRP-3 -72 1 7

MadCAM-1 -74 7 7

TRANCE -75 7 3

GITR L -76 4 4

EGF -78 5 8

TWEAK R -80 5 8

ALK-1 -83 4 6

IL-17 B -84 8 4

Proteins significantly regulated in cortical tissue on 7 days post mTBI

Data are presented as percent 'increase' from sham.

*Up regulated Down regulated*

**Protein mean sem n Protein mean sem n**

VEGF-B 230 56 7 MCSF -21 5 10

SLAM 186 61 6 Fractalkine -28 7 12

IL-17 E 157 37 12 BLC -28 4 12

E-Cadherin 111 32 8 CXCL16 -28 7 12

IL-20 111 33 8 MMP-10 -30 12 11

CD40 L 103 36 9 DLL4 -30 3 11

Galectin-3 87 30 12 IL-15 -32 7 12

CD36 74 26 12 MIG -33 6 12

I-TAC 50 17 11 TCA-3 -34 6 12

GAS 1 40 11 12 Testican 3 -34 11 12

Prolactin 36 13 11 IL-1a -36 6 12

PDGF-AA 32 10 12 IL-6 -42 8 11

MBL-2 31 11 12 E-Selectin -42 3 8

Lungkine 26 8 8 Leptin -44 11 8

Galectin-1 23 6 12 CD30 L -45 8 12

Progranulin 19 4 11 IL-7 Ra -46 5 10

RANTES -48 8 9

GITR -49 5 12

MadCAM-1 -49 8 10

MIP-1a -50 9 12

FAS L -50 5 12

MCP-5 -69 5 5

IL-7 -72 9 9

IL-1ra -74 7 7

Proteins significantly regulated in cortical tissue on 14 days post mTBI

Data are presented as percent 'increase' from sham.

*Up regulated Down regulated*

**Protein mean sem n Protein mean sem n**

BAFF R 99 23 12 Fractalkine -22 4 11

VEGF-B 91 22 10 CXCL16 -24 4 12

CD6 90 26 8 MIG -24 10 12

IL-17 E 87 35 12 Galectin-7 -28 8 12

CD48 87 24 12 IL-1 R4 -32 4 12

IL-20 81 18 9 CD30 L -38 10 12

IL-33 76 15 9 TWEAK R -39 22 12

Meteorin 72 15 11 L-Selectin -42 19 9

Renin 1 72 26 12 MCP-5 -43 14 9

CD27 69 25 12 FAS L -43 6 12

CD40 L 64 26 12 MadCAM-1 -49 8 8

BTC 59 13 12 MIP-2 -50 6 10

E-Cadherin 54 10 9 IL-1 ra -61 12 10

OX 40 Ligand 45 17 11 ALK-1 -63 10 9

OPN 42 11 11 EGF -70 8 11

C5a 36 12 12 G-CSF -75 9 5

MLB-2 25 6 12

Clusterin 21 5 12

Galectin-1 15 3 12

Proteins significantly regulated in cortical tissue on 30 days post mTBI

Data are presented as percent 'increase' from sham.

*Up regulated Down regulated*

**Protein mean sem n Protein mean sem n**

TRANCE 247 63 4 CCL6 -24 3 4

CD40 L 163 40 4 ICAM-1 -30 1 4

CTLA4 119 66 3 MIG -31 12 4

IL-17 E 113 55 4 IFN-g -32 7 3

sFRP-3 96 35 4 IL-2 RA -37 4 4

IFNg R1 88 3 3 DLL4 -41 6 4

ACE 56 20 4 IL-6 -42 11 4

Chemerin 56 6 3 IL-1 ra -44 2 3

VEGF-B 42 6 3 IL-1a -49 4 4

IGFBP-6 27 3 3 OPG -57 11 4

TROY 15 1 3 CRP -57 5 4
